# Ophthalmology Considerations in End-of-Life Care

**DOI:** 10.7759/cureus.94396

**Published:** 2025-10-12

**Authors:** Mendel Shloush, Akiva Eleff, Eric Eleff

**Affiliations:** 1 Orthopedic Surgery, Florida International University Herbert Wertheim College of Medicine, Miami, USA; 2 Emergency Medicine, Ohio University Heritage College of Osteopathic Medicine, Cleveland, USA; 3 Ophthalmology, Louis Stokes Cleveland VA Medical Center, Cleveland, USA

**Keywords:** cataract surgery, hospice, macular degeneration, palliative care, quality of life, retinal detachment, vision

## Abstract

Ophthalmologic interventions can significantly impact quality of life, even in the context of end-of-life care. This paper explores the ethical and clinical considerations for ophthalmologic treatments in hospice care, with a focus on cataract surgery, age-related macular degeneration (ARMD) therapy, retinal detachment (RD) repair, glaucoma, painful blind eye (PBE) management, benign and surface ocular tumors, and corneal or anterior segment diseases. A review of published literature and clinical precedent was conducted to assess the benefits, risks, and practical limitations of ophthalmologic procedures in hospice patients, with an emphasis on quality of life. Cataract surgery has been shown to be cost-effective in hospice settings, offering significant improvements in vision and overall quality of life. Treatment for ARMD, though requiring ongoing injections, can provide benefits within weeks and help sustain visual function. RD repair, while more complex, can substantially restore vision when appropriately managed. Palliative glaucoma interventions, including individualized target intraocular pressures and laser therapy, can minimize treatment burden while maintaining comfort. PBEs may be managed with retrobulbar chlorpromazine or alcohol injections, and enucleation or evisceration may be considered when pain persists. Benign or surface ocular tumors and corneal or anterior segment diseases can often be addressed with conservative, comfort-focused measures to reduce symptoms and preserve quality of life. Ophthalmologic procedures should be considered viable options in end-of-life care when clinically indicated, with careful ethical review. Restoration of vision contributes meaningfully to the quality of life and deserves thoughtful inclusion in care planning.

## Editorial

End-of-life care requires ensuring the mental and physical well-being of patients in the effort to maximize quality of life [1]. This presents many difficult decisions for hospice and palliative care teams, as many comorbid conditions can be treated to improve quality of life but must be weighed against not only common end-of-life protocols but also the potential harm or burden placed on the patient [2]. This often comes down to the ethics of providing treatment, including, but not limited to, considerations of patient safety, survival rates, cost, treatment efficacy, stress of maintaining appointments, transportation, and staff availability for post-care. In this report, we delve into the nuances of common ophthalmologic conditions to delineate when care should be considered within the accepted ethical framework of hospice.

This paper reviews recent and relevant literature, including case studies and peer-reviewed research articles, to evaluate the appropriateness of ophthalmologic procedures in end-of-life care. Sources were identified through targeted searches of major medical databases using terms related to ophthalmologic procedures, hospice care, and ethical decision-making at the end of life. Emphasis was placed on the most up-to-date studies and reviews that offered clinical insights or discussed patient-centered outcomes. Each condition is assessed in the context of hospice eligibility, treatment logistics, anesthesia concerns, expected outcomes, and potential impact on patient quality of life.

In published cases of hospice patients treated with cataract surgery, cost-effectiveness assessments determined that it can be worthwhile to operate in order to increase quality of life [3]. A focal limitation to this procedure is anesthesia clearance, due to the common use of monitored anesthesia care (MAC) [4], which involves planned local anesthesia with sedation and analgesia administered by an anesthesiologist [4]. However, this procedure can, in specific cases, be performed without MAC, and the criteria for allowing MAC can often be met. The postoperative course involves daily eye drops and follow-up ophthalmology visits during the month following the operation. Benefits may be evidenced immediately, with patients able to appreciate color, brightness, and general vision correction. As a relatively low-risk operation, this has even been covered by Medicare for hospice patients on a case-by-case basis.

Another procedure worth consideration is treatment for age-related macular degeneration (ARMD). This involves ocular injections of anti-vascular endothelial growth factor (anti-VEGF) medications every four to six weeks, with a maximum recommended interval of 16 weeks [5]. Among the treatment options, ranibizumab and bevacizumab are the most frequently used anti-VEGF agents in the treatment of neovascular ARMD. Multiple randomized controlled trials have established the efficacy of ranibizumab (Lucentis) for this indication [6-12]. Bevacizumab (Avastin), originally developed for oncology use, is widely used off-label due to its comparable efficacy and lower cost [13,14]. The timeline and dosing are dependent on individualized care and diagnostic testing. This procedure is generally low risk, although complications may arise in patients with recent stroke or myocardial infarction [5]. A limiting factor is the treatment timeline: it may take four weeks to notice a considerable benefit. This becomes relevant if the hospice patient has a short projected lifespan and is treatment naïve. However, if a patient has already received treatment and noted benefit, continuation would be expected to preserve visual gains. Logistical challenges of arranging injections should be weighed against the value of preserving vision.

Retinal detachment (RD) repair also requires critical evaluation. There are three types of RD: rhegmatogenous (RRD), tractional (TRD), and exudative (ERD) [15,16]. RRD is the most common, with risk factors including retinal tears, lattice degeneration, prior ocular surgery, family history, and high myopia. TRD is often associated with proliferative diabetic retinopathy, vitreoretinopathy, trauma, and retinal vein occlusion. RRD and TRD are particularly relevant to elderly and end-of-life care patients. Common curative procedures include pars plana vitrectomy, scleral buckle, and pneumatic retinopexy. The most common complication is proliferative vitreoretinopathy, occurring in an estimated 5-10% of patients and typically presenting within 30-45 days postoperatively [17-24]. Risk factors for this complication can be managed through collaboration with a retinal specialist and appropriate follow-up, although such follow-up may not be routinely covered by hospice agencies and can present a logistical barrier. Concerns related to MAC, as previously discussed, can sometimes be addressed through modified anesthesia plans, such as performing the procedure under local or topical anesthesia when feasible. However, in patients with advanced or terminal illness, these considerations remain significant, as sedation may worsen delirium or lead to unwanted side effects and must therefore be carefully weighed against potential benefits. While there is little published research on RD repair outcomes in end-of-life care, there are documented cases of substantial visual improvement, often to 20/40 or better in the affected eye.

In end-of-life care, glaucoma management focuses on comfort, minimizing treatment burden, and maintaining quality of life rather than long-term vision preservation [25,26]. The American Academy of Ophthalmology currently recommends individualized goals, with less aggressive pressure control when disease is stable or life expectancy is limited [25,26]. Target intraocular pressure (IOP) is usually set about 25% below baseline, with lower targets for advanced disease [25-28]. In palliative contexts, higher IOP targets and simplified drop regimens can reduce side effects while maintaining comfort [28].

Laser trabeculoplasty, particularly selective laser trabeculoplasty (SLT), is a safe, office-based option that lowers IOP and lessens medication dependence [25-27,29]. SLT has a favorable safety profile and may be considered when adherence is difficult or when minimizing daily medications is a priority [25-27,29,30]. High-quality data on palliative glaucoma management are limited, so treatment should be guided by comfort, practicality, and shared decision-making between ophthalmology and palliative teams [25,29].

A painful blind eye (PBE) causes significant discomfort and reduced quality of life in patients with no visual potential [31]. There are no formal guidelines, so management is individualized [31]. Medical therapy with topical steroids, cycloplegics, and hypotensive agents offers partial relief in about 40% of cases, but many patients require further intervention [32]. When pain persists, retrobulbar injections of alcohol or chlorpromazine can provide effective palliation by blocking the ciliary nerves and lowering IOP [33-35]. Pain relief occurs in roughly 40% of cases, though some patients need repeat treatment or later eye removal [33,34]. Chlorpromazine may offer longer relief, lasting at least three months in up to 80% of cases [34]. Retrobulbar alcohol is more likely to cause orbital inflammation and fibrosis, which can complicate later surgery [36-38]. Chlorpromazine may cause transient periocular inflammation or eyelid swelling, but these usually resolve within weeks [33,39,40]. Chlorpromazine is preferred when future enucleation or evisceration might be needed due to its lower risk of chronic inflammation [36,37]. Enucleation or evisceration remains the definitive treatment, providing complete and lasting pain relief and eliminating the need for systemic analgesics [32,41]. A stepwise approach is recommended, escalating from medical therapy to minimally invasive options and finally to surgery when indicated [31,32].

Palliative management of benign and surface tumors such as basal cell carcinoma (BCC), squamous cell carcinoma (SCC), and conjunctival intraepithelial neoplasia (CIN) focuses on comfort, symptom relief, and reducing treatment burden in patients with limited life expectancy [42,43]. For low-risk BCC and SCC in situ, topical agents such as imiquimod and fluorouracil can provide reasonable local control with minimal invasiveness, although recurrence is more common than after surgical excision [42-44]. Photodynamic therapy offers another conservative approach, especially for superficial BCC or SCC, and yields good cosmetic results [44-46].

When surgery is not an option, radiation therapy may be used for palliation to control symptoms such as bleeding or pain, though it carries a higher risk of long-term side effects [45,47]. For conjunctival and ocular surface lesions such as CIN and ocular surface squamous neoplasia, topical mitomycin C, interferon alfa-2b, or fluorouracil are effective and well-tolerated alternatives to excision [47,48]. Supportive care should include wound management, analgesia, and infection or odor control when needed [49]. Shared decision-making remains essential to align therapy with the patient’s goals and overall prognosis.

Management of Fuchs endothelial dystrophy, bullous keratopathy, and exposure keratopathy focuses on comfort and symptom relief when surgery is not feasible or aligned with patient goals [50,51]. For Fuchs dystrophy, symptom control uses frequent lubrication, hypertonic saline, and bandage contact lenses [50]. Keratoplasty is curative but rarely suitable in palliative care, and experimental therapies are not standard [52-55,56]. Bullous keratopathy is managed with lubrication, hypertonic saline, and bandage lenses, with conjunctival flap, amniotic membrane transplantation, or anterior stromal puncture for refractory pain [50,57-59]. Pressure patching is not recommended [50,57]. Exposure keratopathy relies on lubrication, moisture chambers, and bandage lenses, with tarsorrhaphy or adjunctive therapies such as amniotic membrane grafts, serum tears, or platelet lysate for severe or non-healing cases (Table 1) [50,51,59,60].

**Table 1:** Key ophthalmologic conditions and palliative interventions in hospice care ARMD, age-related macular degeneration; BCC, basal cell carcinoma; CIN, conjunctival intraepithelial neoplasia; IOP, intraocular pressure; PBE, painful blind eye; RD, retinal detachment; RRD, rhegmatogenous retinal detachment; SCC, squamous cell carcinoma; SLT, selective laser trabeculoplasty; TRD, tractional retinal detachment

Condition/disease	Palliative/comfort-focused intervention	Notes/considerations
Cataract	Surgery (phacoemulsification)	Low-risk; improves vision quickly; follow-up drops required; Medicare may cover in select hospice cases
ARMD	Anti-VEGF injections (ranibizumab and bevacizumab)	Four- to six-week intervals; continuation preserves vision; benefit may take weeks; assess patient prognosis
RD (RRD and TRD)	Pars plana vitrectomy, scleral buckle, pneumatic retinopexy	Consider anesthesia risk, postoperative recovery, logistical feasibility; can restore vision to 20/40 in select patients
Glaucoma	Palliative target IOP (~25% below baseline), simplified drops, SLT	Focus on comfort and minimizing burden rather than aggressive IOP control
PBE	Retrobulbar chlorpromazine or alcohol injections; enucleation/evisceration if needed	Stepwise escalation from meds → injection → surgery; shared decision-making essential
Benign/surface tumors (BCC, SCC, and CIN)	Topical therapy (imiquimod, fluorouracil, mitomycin C, interferon alfa-2b); photodynamic therapy; palliative radiation if surgery not feasible	Aim to control symptoms, reduce burden, preserve comfort; recurrence possible with topical therapy
Fuchs endothelial dystrophy	Lubrication, hypertonic saline, bandage, contact lenses	Keratoplasty rarely appropriate in palliative care; focus on symptom relief
Bullous keratopathy	Lubrication, hypertonic saline, bandage lenses; conjunctival flap or anterior stromal puncture for refractory pain	Surgery considered only if aligned with the goals of comfort
Exposure keratopathy	Lubrication, moisture chambers, bandage lenses; tarsorrhaphy or amniotic membrane graft for severe cases	Symptom-focused; shared decision-making to align with patient goals

Ethical decision-making in palliative ophthalmology emphasizes proportionality, ensuring that the benefits of interventions outweigh the burdens, particularly in patients with limited life expectancy. Patient goals should guide all management decisions, with comfort, quality of life, and functional priorities taking precedence over aggressive disease control. Shared decision-making with patients and caregivers is essential to align treatment strategies with individual preferences. In hospice care, this framework helps determine whether ophthalmologic procedures, such as vision restoration or preservation, can meaningfully enhance quality of life, independence, and emotional well-being during the final stage of life.

Existing evidence supports cataract surgery, anti-VEGF therapy for ARMD, and RD repair in carefully selected hospice patients when benefits outweigh physical and psychological burdens. Palliative glaucoma management with comfort-focused pressure targets and laser therapy, retrobulbar injections for PBEs, conservative treatment of surface tumors, and lubrication or tarsorrhaphy for corneal and anterior segment diseases can also improve quality of life. For RD, clinicians should weigh procedure duration, anesthesia needs, and postoperative recovery against the patient’s prognosis and tolerance for procedural stress. In some patients, restoring vision may reduce confusion or delirium, while in others, the distress of surgery could outweigh the benefit. Individualized decision-making ensures all interventions align with patient goals and priorities.

Cataract surgery is straightforward and low risk and offers quick results: brighter, clearer vision that patients notice right away. The follow-up, involving eye drops and a few check-ups, is doable in most cases, and Medicare’s willingness to cover it in specific hospice scenarios shows its value. Anti-VEGF therapy for ARMD helps keep vision stable, especially for those already benefiting, but its four- to six-week timeline might not work for patients with a very short prognosis. RD repair, though trickier, can dramatically improve vision, often to 20/40 or better, and risks like proliferative vitreoretinopathy can be handled with specialist input.

These treatments align with hospice’s core aim of ensuring comfort and independence. Research consistently finds that good vision ties more closely to quality of life than many other major health issues [61-66]. That said, practical hurdles such as getting patients to appointments or arranging follow-up care cannot be ignored. We also need more studies on long-term results and costs, specifically for hospice patients. In the end, the choice to pursue these procedures should come from a patient’s own goals, their expected time left, and what’s realistically achievable, ensuring their remaining time is as rich and fulfilling as possible.

Vision plays a significant role in a patient’s sense of independence and overall quality of life, even in the context of end-of-life care. While hospice and palliative care traditionally focus on comfort and symptom management, they also prioritize quality of life and should not exclude interventions that can meaningfully improve a patient’s daily function and well-being. Cataract surgery, anti-VEGF therapy for ARMD, and RD repair each have the potential to restore or preserve vision and may be reasonable to offer in select hospice patients. Palliative glaucoma treatment, PBE management, conservative therapy for surface tumors, and lubrication or tarsorrhaphy for corneal and anterior segment diseases can also improve comfort and quality of life. These decisions should be made through a careful ethical lens, considering patient goals, expected prognosis, procedural burden, and potential for benefit. As the field continues to evolve, further research and dialogue between palliative and ophthalmologic teams will be essential in guiding thoughtful, patient-centered care.

## References

[1] Rummans TA, Bostwick JM, Clark MM (2000). Maintaining quality of life at the end of life. Mayo Clin Proc.

[2] Eleff A (2022). Harm versus burden. Am J Hosp Palliat Care.

[3] Nunn JG, Shreve S, Scott IU, Pantanelli SM (2016). Cataract surgery in hospice patients: pros, cons, and cost-effectiveness. J Palliat Med.

[4] Sohn HM, Ryu JH (2016). Monitored anesthesia care in and outside the operating room. Korean J Anesthesiol.

[5] Fleckenstein M, Keenan TD, Guymer RH (2021). Age-related macular degeneration. Nat Rev Dis Primers.

[6] Gaudreault J, Fei D, Rusit J, Suboc P, Shiu V (2005). Preclinical pharmacokinetics of ranibizumab (rhuFabV2) after a single intravitreal administration. Invest Ophthalmol Vis Sci.

[7] Brown DM, Kaiser PK, Michels M (2006). Ranibizumab versus verteporfin for neovascular age-related macular degeneration. N Engl J Med.

[8] Heier JS, Boyer DS, Ciulla TA (2006). Ranibizumab combined with verteporfin photodynamic therapy in neovascular age-related macular degeneration: year 1 results of the FOCUS Study. Arch Ophthalmol.

[9] Rosenfeld PJ, Brown DM, Heier JS, Boyer DS, Kaiser PK, Chung CY, Kim RY (2006). Ranibizumab for neovascular age-related macular degeneration. N Engl J Med.

[10] Fung AE, Lalwani GA, Rosenfeld PJ (2007). An optical coherence tomography-guided, variable dosing regimen with intravitreal ranibizumab (Lucentis) for neovascular age-related macular degeneration. Am J Ophthalmol.

[11] Regillo CD, Brown DM, Abraham P, Yue H, Ianchulev T, Schneider S, Shams N (2008). Randomized, double-masked, sham-controlled trial of ranibizumab for neovascular age-related macular degeneration: PIER Study year 1. Am J Ophthalmol.

[12] Schmidt-Erfurth U, Eldem B, Guymer R (2011). Efficacy and safety of monthly versus quarterly ranibizumab treatment in neovascular age-related macular degeneration: the EXCITE study. Ophthalmology.

[13] Cohen MH, Gootenberg J, Keegan P, Pazdur R (2007). FDA drug approval summary: bevacizumab (Avastin®) plus Carboplatin and Paclitaxel as first-line treatment of advanced/metastatic recurrent nonsquamous non-small cell lung cancer. Oncologist.

[14] Lushchyk T, Amarakoon S, Martinez-Ciriano JP, van den Born LI, Baarsma GS, Missotten T (2013). Bevacizumab in age-related macular degeneration: a randomized controlled trial on the effect of injections every 4 weeks, 6 weeks and 8 weeks. Acta Ophthalmol.

[15] Blair K, Czyz CN (2025). Retinal detachment. StatPearls [Internet].

[16] Mishra C, Tripathy K (2025). Retinal traction detachment. StatPearls [Internet].

[17] Charteris DG, Sethi CS, Lewis GP, Fisher SK (2002). Proliferative vitreoretinopathy-developments in adjunctive treatment and retinal pathology. Eye (Lond).

[18] Speicher MA, Fu AD, Martin JP, von Fricken MA (2000). Primary vitrectomy alone for repair of retinal detachments following cataract surgery. Retina.

[19] Duquesne N, Bonnet M, Adeleine P (1996). Preoperative vitreous hemorrhage associated with rhegmatogenous retinal detachment: a risk factor for postoperative proliferative vitreoretinopathy?. Graefes Arch Clin Exp Ophthalmol.

[20] Greven CM, Sanders RJ, Brown GC (1992). Pseudophakic retinal detachments. Anatomic and visual results. Ophthalmology.

[21] Girard P, Mimoun G, Karpouzas I, Montefiore G (1994). Clinical risk factors for proliferative vitreoretinopathy after retinal detachment surgery. Retina.

[22] Gartry DS, Chignell AH, Franks WA, Wong D (1993). Pars plana vitrectomy for the treatment of rhegmatogenous retinal detachment uncomplicated by advanced proliferative vitreoretinopathy. Br J Ophthalmol.

[23] Bonnet M, Fleury J, Guenoun S, Yaniali A, Dumas C, Hajjar C (1996). Cryopexy in primary rhegmatogenous retinal detachment: a risk factor for postoperative proliferative vitreoretinopathy?. Graefes Arch Clin Exp Ophthalmol.

[24] Heimann H, Bornfeld N, Friedrichs W, Helbig H, Kellner U, Korra A, Foerster MH (1996). Primary vitrectomy without scleral buckling for rhegmatogenous retinal detachment. Graefes Arch Clin Exp Ophthalmol.

[25] Gedde SJ, Vinod K, Wright MM (2021). Primary Open-Angle Glaucoma Preferred Practice Pattern®. Ophthalmology.

[26] Weinreb RN, Aung T, Medeiros FA (2014). The pathophysiology and treatment of glaucoma: a review. JAMA.

[27] Glaucoma summary benchmarks - 2024. https://www.aao.org/education/summary-benchmark-detail/glaucoma-summary-benchmarks-2020.

[28] Sihota R, Angmo D, Ramaswamy D, Dada T (2018). Simplifying "target" intraocular pressure for different stages of primary open-angle glaucoma and primary angle-closure glaucoma. Indian J Ophthalmol.

[29] Rolim-de-Moura CR, Paranhos A Jr, Loutfi M, Burton D, Wormald R, Evans JR (2022). Laser trabeculoplasty for open-angle glaucoma and ocular hypertension. Cochrane Database Syst Rev.

[30] Reporting clinical endpoints in studies of minimally invasive glaucoma surgery - 2025. Ophthalmology.

[31] Parra-Tanoux D, Dussan-Vargas MP, Escandon MG (2023). Painful-blind eye: a forgotten palliative care. Indian J Ophthalmol.

[32] Idowu OO, Ashraf DC, Kalin-Hajdu E, Ryan MC, Kersten RC, Vagefi MR (2019). Efficacy of care for blind painful eyes. Ophthalmic Plast Reconstr Surg.

[33] Galindo-Ferreiro A, Akaishi P, Cruz A (2016). Retrobulbar injections for blind painful eyes: a comparative study of retrobulbar alcohol versus chlorpromazine. J Glaucoma.

[34] Chen TC, Ahn Yuen SJ, Sangalang MA, Fernando RE, Leuenberger EU (2002). Retrobulbar chlorpromazine injections for the management of blind and seeing painful eyes. J Glaucoma.

[35] Kumar CM, Dowd TC, Hawthorne M (2006). Retrobulbar alcohol injection for orbital pain relief under difficult circumstances: a case report. Ann Acad Med Singap.

[36] Eftekhari K, Shindler KS, Lee V, Dine K, Eckstein LA, Vagefi MR (2016). Histologic evidence of orbital inflammation from retrobulbar alcohol and chlorpromazine injection: a clinicopathologic study in human & rat orbits. Ophthalmic Plast Reconstr Surg.

[37] Cotliar JM, Shields CL, Meyer DR (2008). Chronic orbital inflammation and fibrosis after retrobulbar alcohol and chlorpromazine injections in a patient with choroidal melanoma. Ophthalmic Plast Reconstr Surg.

[38] Bialer OY, Saindane AM, Newman NJ (2014). Orbital MRI appearance after remote retrobulbar alcohol injection. Ophthalmic Plast Reconstr Surg.

[39] McCulley TJ, Kersten RC (2006). Periocular inflammation after retrobulbar chlorpromazine (thorazine) injection. Ophthalmic Plast Reconstr Surg.

[40] Hauck MJ, Lee HH, Timoney PJ, Shoshani Y, Nunery WR (2012). Neurotrophic corneal ulcer after retrobulbar injection of chlorpromazine. Ophthalmic Plast Reconstr Surg.

[41] Shah-Desai SD, Tyers AG, Manners RM (2000). Painful blind eye: efficacy of enucleation and evisceration in resolving ocular pain. Br J Ophthalmol.

[42] Nehal KS, Bichakjian CK (2018). Update on keratinocyte carcinomas. N Engl J Med.

[43] Algarin YA, Jambusaria-Pahlajani A, Ruiz E, Patel VA (2023). Advances in topical treatments of cutaneous malignancies. Am J Clin Dermatol.

[44] Altun E, Schwartzman G, Cartron AM, Khachemoune A (2021). Beyond Mohs surgery and excisions: a focused review of treatment options for subtypes of basal cell carcinoma. Dermatol Ther.

[45] Firnhaber JM (2020). Basal cell and cutaneous squamous cell carcinomas: diagnosis and treatment. Am Fam Physician.

[46] Thomson J, Hogan S, Leonardi-Bee J, Williams HC, Bath-Hextall FJ (2020). Interventions for basal cell carcinoma of the skin. Cochrane Database Syst Rev.

[47] Hooper J, Shao K, Feng PW, Falcone M, Feng H (2024). Periocular and ocular surface nonmelanoma skin cancer. Clin Dermatol.

[48] Kim JW, Abramson DH (2008). Topical treatment options for conjunctival neoplasms. Clin Ophthalmol.

[49] Oprea Y, Deutsch A, McLellan B, Markova A (2025). Palliative oncodermatology: management of malignancy-related cutaneous symptoms in the palliative care setting. J Am Acad Dermatol.

[50] Cornea/external disease summary benchmarks - 2024. https://www.aao.org/education/summary-benchmark-detail/cornea-external-disease-summary-benchmarks-2020.

[51] Bartlett AH, Bartlett JD (2015). Ophthalmic procedures for treatment of advanced ocular surface diseases. Optom Vis Sci.

[52] Patel SV, Gupta N, Bhogal M (2025). Delphi-based global consensus on Fuchs endothelial corneal dystrophy. An Endothelial Keratoplasty Learners Group initiative. Am J Ophthalmol.

[53] Vedana G, Villarreal G Jr, Jun AS (2016). Fuchs endothelial corneal dystrophy: current perspectives. Clin Ophthalmol.

[54] Soh YQ, Peh GS, Mehta JS (2018). Evolving therapies for Fuchs' endothelial dystrophy. Regen Med.

[55] Cheong N, Chui SW, Poon SH, Wong HL, Shih KC, Chan YK (2024). Emerging treatments for corneal endothelium decompensation — a systematic review. Graefes Arch Clin Exp Ophthalmol.

[56] Kinoshita S, Koizumi N, Ueno M (2018). Injection of cultured cells with a ROCK inhibitor for bullous keratopathy. N Engl J Med.

[57] Siu GD, Young AL, Jhanji V (2014). Alternatives to corneal transplantation for the management of bullous keratopathy. Curr Opin Ophthalmol.

[58] Cataracts. Cicinelli MV, Buchan JC, Nicholson M, Varadaraj V, Khanna RC (2023). Cataracts. Lancet.

[59] Dang DH, Riaz KM, Karamichos D (2022). Treatment of non-infectious corneal injury: review of diagnostic agents, therapeutic medications, and future targets. Drugs.

[60] Tsai CY, Huang WL, Yang SC (2025). Human platelet lysate treatment for exposure keratopathy: an in vivo confocal microscopy and anterior segment OCT study. Invest Ophthalmol Vis Sci.

[61] Purola P, Koskinen S, Uusitalo H (2023). Impact of vision on generic health-related quality of life - a systematic review. Acta Ophthalmol.

[62] Chia EM, Wang JJ, Rochtchina E, Smith W, Cumming RR, Mitchell P (2004). Impact of bilateral visual impairment on health-related quality of life: the Blue Mountains Eye Study. Invest Ophthalmol Vis Sci.

[63] Esteban JJ, Martínez MS, Navalón PG, Serrano OP, Patiño JR, Purón ME, Martínez-Vizcaíno V (2008). Visual impairment and quality of life: gender differences in the elderly in Cuenca, Spain. Qual Life Res.

[64] Nutheti R, Shamanna BR, Nirmalan PK, Keeffe JE, Krishnaiah S, Rao GN, Thomas R (2006). Impact of impaired vision and eye disease on quality of life in Andhra Pradesh. Invest Ophthalmol Vis Sci.

[65] Purola PK, Nättinen JE, Ojamo MU, Koskinen SV, Rissanen HA, Sainio PR, Uusitalo HM (2021). Prevalence and 11-year incidence of common eye diseases and their relation to health-related quality of life, mental health, and visual impairment. Qual Life Res.

[66] Taipale J, Mikhailova A, Ojamo M (2019). Low vision status and declining vision decrease health-related quality of life: results from a nationwide 11-year follow-up study. Qual Life Res.

